# A novel standardized algorithm using SPECT/CT evaluating unhappy patients after unicondylar knee arthroplasty– a combined analysis of tracer uptake distribution and component position

**DOI:** 10.1186/s12880-015-0053-4

**Published:** 2015-03-20

**Authors:** Basil Suter, Enrique Testa, Patrick Stämpfli, Praveen Konala, Helmut Rasch, Niklaus F Friederich, Michael T Hirschmann

**Affiliations:** Department of Orthopaedic Surgery and Traumatology, Kantonsspital Baselland (Bruderholz, Liestal, Laifen), CH-4101, Bruderholz, Switzerland; Department of Radiology, Plymouth, UK; Institute for Radiology and Nuclear Medicine, Kantonsspital Baselland, Bruderholz, CH-4101 Switzerland

**Keywords:** Knee, SPECT/CT, SPECT-CT, Unicondylar knee arthroplasty, Component position, Localization scheme, 3D-CT, Unicompartmental knee replacement, Bone

## Abstract

**Background:**

The introduction of a standardized SPECT/CT algorithm including a localization scheme, which allows accurate identification of specific patterns and thresholds of SPECT/CT tracer uptake, could lead to a better understanding of the bone remodeling and specific failure modes of unicondylar knee arthroplasty (UKA). The purpose of the present study was to introduce a novel standardized SPECT/CT algorithm for patients after UKA and evaluate its clinical applicability, usefulness and inter- and intra-observer reliability.

**Methods:**

Tc-HDP-SPECT/CT images of consecutive patients (median age 65, range 48–84 years) with 21 knees after UKA were prospectively evaluated. The tracer activity on SPECT/CT was localized using a specific standardized UKA localization scheme. For tracer uptake analysis (intensity and anatomical distribution pattern) a 3D volumetric quantification method was used. The maximum intensity values were recorded for each anatomical area. In addition, ratios between the respective value in the measured area and the background tracer activity were calculated. The femoral and tibial component position (varus-valgus, flexion-extension, internal and external rotation) was determined in 3D-CT. The inter- and intraobserver reliability of the localization scheme, grading of the tracer activity and component measurements were determined by calculating the intraclass correlation coefficients (ICC).

**Results:**

The localization scheme, grading of the tracer activity and component measurements showed high inter- and intra-observer reliabilities for all regions (tibia, femur and patella). For measurement of component position there was strong agreement between the readings of the two observers; the ICC for the orientation of the femoral component was 0.73-1.00 (intra-observer reliability) and 0.91-1.00 (inter-observer reliability). The ICC for the orientation of the tibial component was 0.75-1.00 (intra-observer reliability) and 0.77-1.00 (inter-observer reliability).

**Conclusions:**

The SPECT/CT algorithm presented combining the mechanical information on UKA component position, alignment and metabolic data is highly reliable and proved to be a valuable, consistent and useful tool for analysing postoperative knees after UKA. Using this standardized approach in clinical studies might be helpful in establishing the diagnosis in patients with pain after UKA.

## Background

Unicondylar knee arthroplasty (UKA) is considered to be a successful treatment in patients with unicompartmental osteoarthritis of the knee joint [1-5]. However, a considerable number of patients after UKA suffer from persistent or recurrent pain. A variety of causes and failure modes of UKA such as impingement, malposition, malalignment, tibial component subsidence, infection, osteonecrosis, fractures or aseptic loosening have been described [6-11]. To guide an optimal treatment in these challenging patients the cause or origin of the pain has to be accurately identified. Only then revision surgery can improve the patient’s symptoms [12-15]. The standard diaqnostics in patients with pain after UKA include a detailed medical history, a thorough clinical examination, conventional radiographs and planar or 3D scintigraphy (SPECT) [16-19]. MRI is more frequently used for evaluation of UKA prosthesis bone interface [20,21]. However, due to the metal artifacts caused by the UKA its clinical value is questionable. However, metal artifact reduction software and improved imaging protocols will lead to a better diagnostic quality of MRI in future [22].

Combining the advantages of CT and SPECT a hybrid imaging modality (SPECT/CT) offers the combined assessment of the bone tracer activity of the joint, mechanical alignment and UKA component position [23-27]. SPECT/CT is able to accurately allocate the metabolic activity in a region of interest to specific anatomical areas and gain a more profound insight into the osseointegration process after UKA, which happens in the bone-prosthesis interface [24,25]. After UKA, whether cemented or uncemented the prosthesis is fixed directly or via cement to the bone. This osseointegration is not understood well enough, when it comes to UKA.

Due to its imaging characteristics SPECT/CT has been used to evaluate patients after total knee arthroplasty, anterior cruciate ligament reconstruction, cartilage surgery or high tibial osteotomies [23-25,27-35]. Although SPECT/CT has been proposed as diagnostic adjunct in patients with pain after UKA, neither a standardized algorithm for evaluating patients after UKA nor a specific localization scheme characterizing tracer activity in these patients have been reported so far. In summary SPECT/CT has the potential to lead to a better understanding of the specific UKA failure modes. The purpose of the present study was to introduce a standardized SPECT/CT algorithm for patients after UKA and evaluate its clinical applicability, usefulness and inter- and intra-observer reliability.

## Methods

^99m^Tc-HDP-SPECT/CT images of consecutive patients (median age 65, range 48–84 years, 12 males, 9 females) with 21 knees after UKA were prospectively collected and evaluated. The mean follow-up was 15 ± 4 months. The study was approved by the Institutional Review Board (EKBB). Written informed consent for participation in the study was obtained from participants.

SPECT/CT was performed using a hybrid system (Symbia T16, Siemens, Erlangen, Germany), which is equipped with a dual-head gamma camera with an integrated 16-slice CT scanner (collimation of 16x0.75-mm).

All patients received a commercial 500 MBq, ^99m^Tc-HDP injection (Malinckrodt, Wellerau, Switzerland). Planar scintigraphic images were taken in three phases, the perfusion phase (immediately after injection), the soft tissue phase (from 1 to 5 minutes after injection) and the delayed phase (from 2 hours after injection). SPECT/CT was performed with a matrix size of 128x128, an angle step of 32, and a time per frame of 25 s two-three hours after injection. The CT protocol was modified according to the Imperial Knee Protocol, which is a CT protocol that includes Ct scans of 0.7 mm slices of the knee and 3 mm slices of the hip and ankle joints [36]. This protocol allows accurate determination of mechanical alignment and UKA component position in 3D.

Data were processed by interactive reconstruction on a work platform (Syngo, Siemens Erlangen, Germany). Images were displayed in orthogonal axial, coronal and sagittal planes.

### Tracer uptake analysis

SPECT/CT tracer activity was localized using a specific standardized UKA localization scheme, which was modified from the previously introduced TKA localization scheme [27]. For tracer uptake analysis (intensity and anatomical distribution pattern) the 3D reconstructed datasets of the delayed SPECT/CT images were used. Each anatomical area was measured in terms of SPECT/CT tracer uptake values [24]. The maximum intensity values were recorded for each anatomical area. In addition, ratios between the respective value in the measured area and the background tracer activity (proximal mid-shaft of the femur) were calculated.

**Figure 1:**
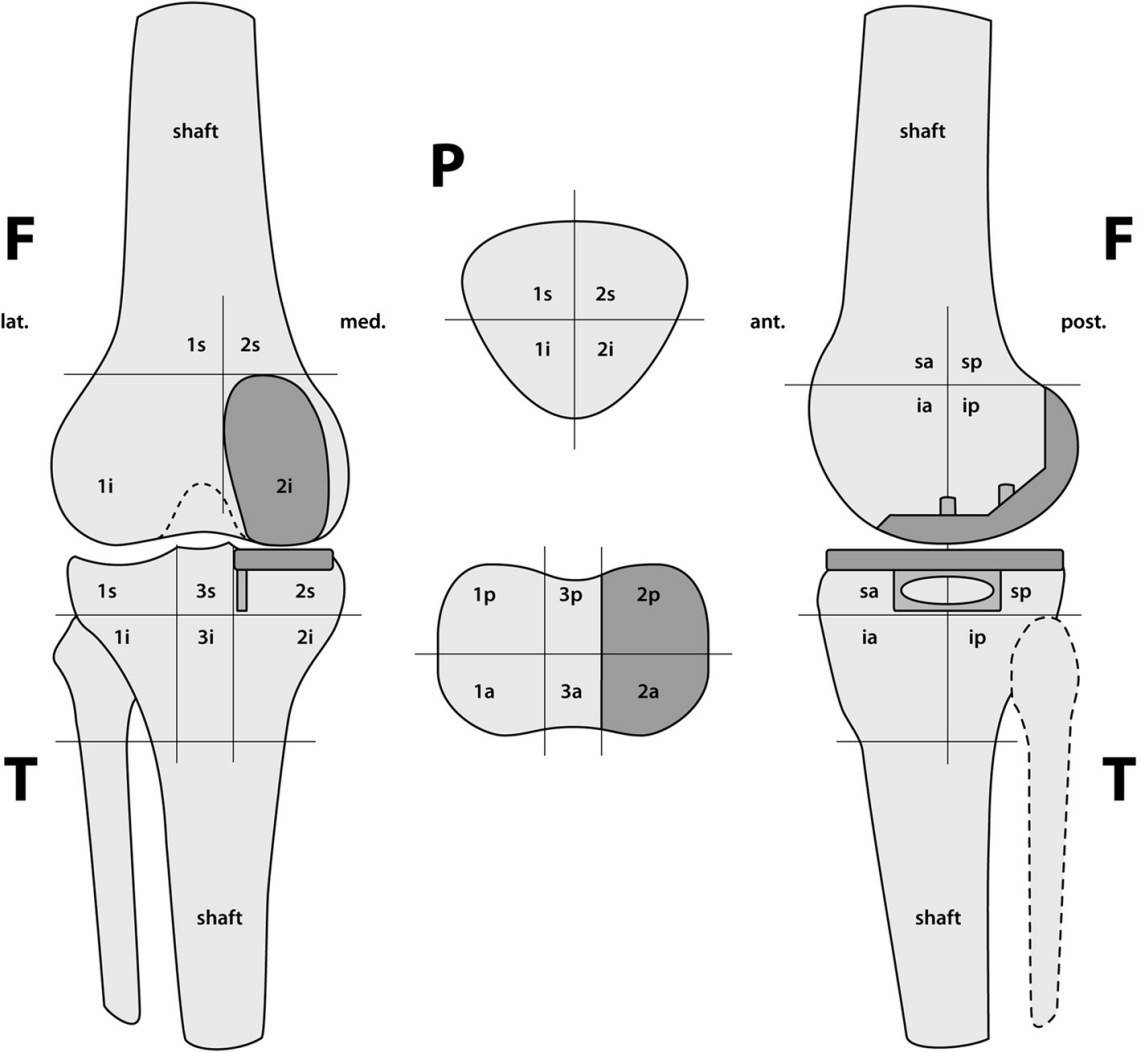
**The localization scheme for the Tc-99 m HDP tracer activity in patients after UKA (Femur = F, Tibia = T, Patella = P, 1 = medial, 2 = lateral, 3 = central around stem, a = anterior, p = posterior, i = inferior, s = superior, shaft, tip and tubercle).**

The tracer activity was quantified in 3D and volumetrically as previously described in (Figure 1) [24]. The quantification is done by the software using anatomically specified voxel boxes. The intensity values of bone tracer uptake are obtained from the system’s raw data.

One senior orthopaedic surgeon (MTH) with over 12 years experience in orthopaedic surgery and 10 years experience in imaging analysis and one musculoskeletal radiologist and nuclear medicine physician (HR) over 10 years experience in SPECT and CT analysis assessed the SPECT/CTs in all patients twice with two weeks intervals between measurements in random order. Both were blinded to results from previous observations.

### Measurement of femoral and tibial component position

**Figure 2:**
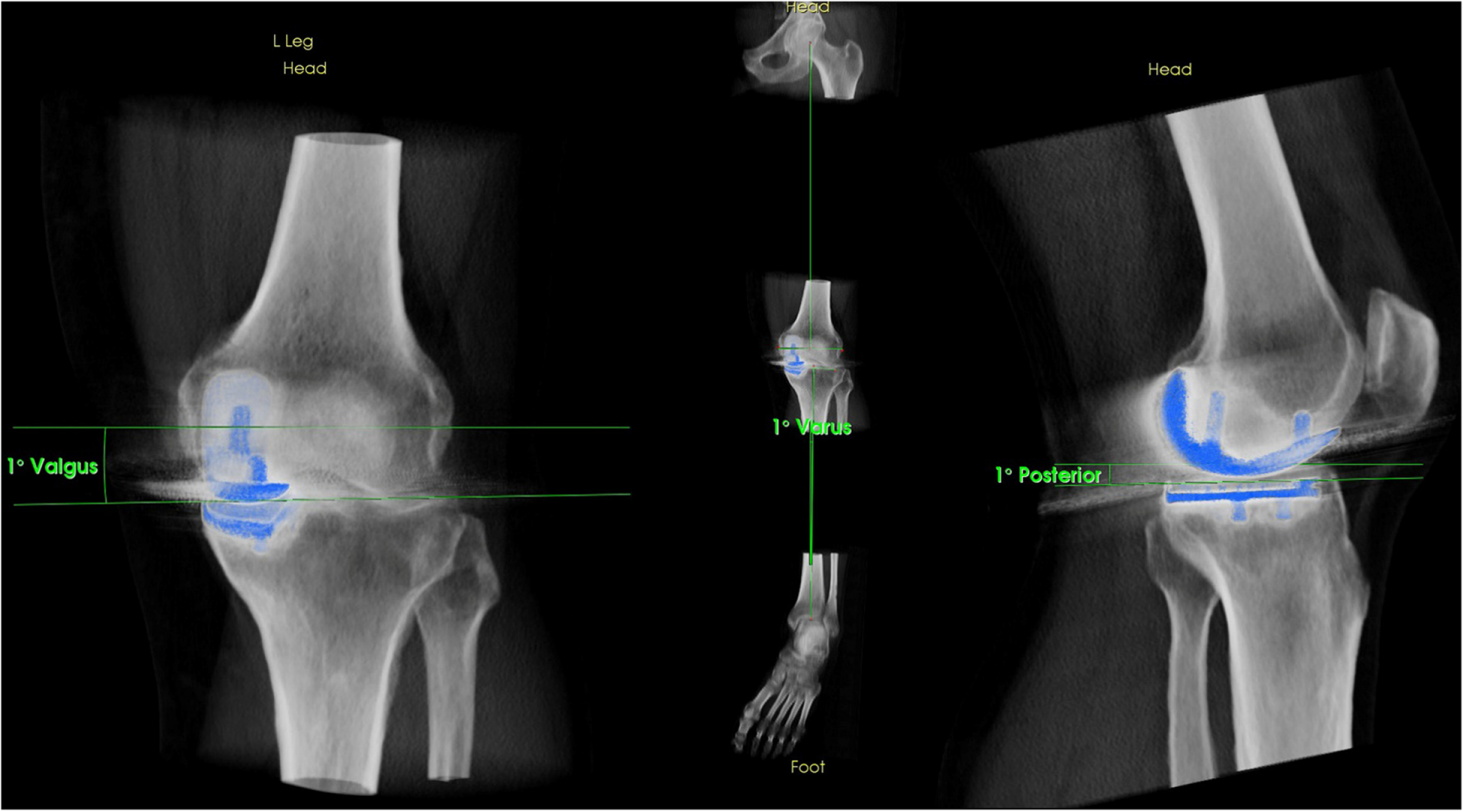
**Determination of UKA component position in 3D reconstructed CT images using a customized software.**

**Figure 3:**
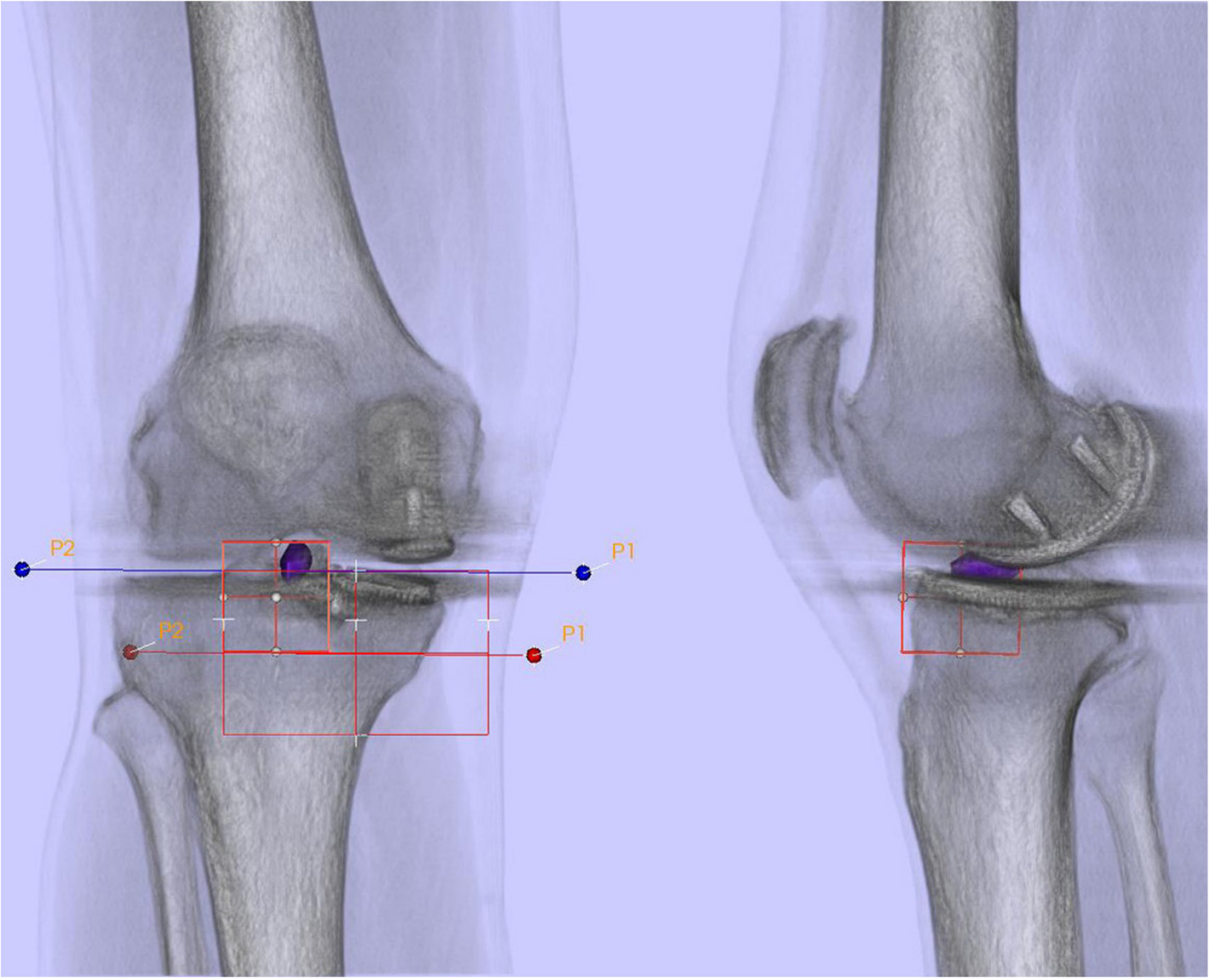
**Bone tracer uptake quantification of a SPECT/CT in standardized anatomical areas in an asymptomatic patient one year after UKA.**

The position of the femoral and tibial UKA components was assessed using a customized software, which is able to reconstruct three-dimensional images from CT data. These 3D models enabled the observer to perform measurements in terms of angles (in degrees) and distances (in mm). The femoral component position (varus-valgus, flexion-extension, internal and external rotation) was determined in relation to the transepicondylar axis and the mechanical femoral axis. The tibial component position (varus-valgus, anterior-posterior slope, internal and external rotation) was determined with regards to the tibial posterior condylar axis and the anatomical and mechanical femoral shaft axis (Figure 2). In addition, a possible femoral and tibial rotational mismatch was determined (Figure 3). The same observers performed these measurements in random order. Both observers performed the measurements twice and were blinded to results from previous observations.

### Statistical analysis

Data were analyzed using SPSS 17.0 (SPSS, Chicago, U.S.A.). Sample size was calculated according to the reported estimates for reliability studies using intraclass correlation coefficients (ICCs) [37].

The inter- and intra-observer reliability of the localization scheme, grading of the tracer activity and UKA component measurements were determined by calculating the intraclass correlation coefficients (ICC). An ICC value of 1 indicates perfect reliability, 0.81 to 1 very good reliability and 0.61-0.80 good reliability [37]. The median inter- and intra-observer differences in component measurements were also calculated.

## Results

**Figure 4:**
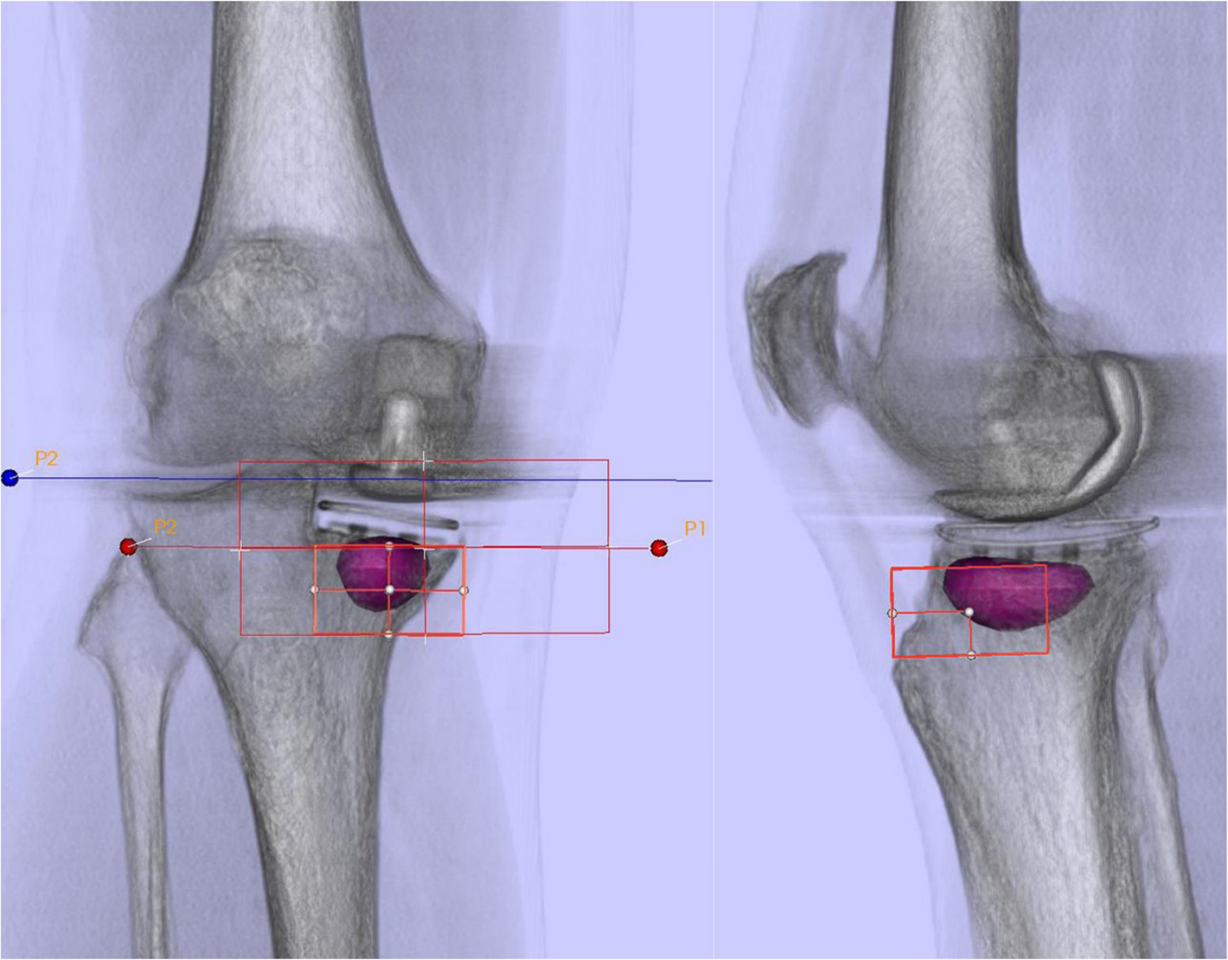
**Bone tracer uptake quantification of a SPECT/CT in standardized anatomical areas in a symptomatic patient with tibial loosening five year after UKA.**

All regions with tracer uptake were present in the localization scheme and the tracer uptake could be located to specific anatomical regions in all cases (Figures 4). The SPECT/CT localization scheme showed high inter- and intra-observer reliabilities for all regions (tibia, femur and patella). The ICCs are presented in Table 1. Figures 3 and 4 show the application of the localisation scheme in two typical patients.

**Table 1:** **Inter- and intra-observer reliability (intra class correlation- ICC) of 99mTc-HDP-SPECT/CT activity using the localization and tracer uptake intensity analysis for the tibia, the femur and the patella**

			**Intrarater-reliability**				**Interrater-reliability**	
**No**	**Location**	**Type**	**U1**		**U2**		**U1-U2**	
			**Absolute**	**Relative**	**Absolute**	**Relative**	**Absolute**	**Relative**
1	F2ip	Mean	1.00	0.85	1.00	0.96	1.00	0.96
2	F1ip	Mean	0.99	0.87	1.00	0.96	0.98	0.93
3	F2ia	Mean	0.98	0.89	0.99	0.99	0.99	0.91
4	F1ia	Max	0.97	0.73	0.94	0.95	0.98	0.96
5	F2sa	Max	0.95	0,88	0.99	0.99	0.97	0.95
6	F1sa	Max	0.89	0.81	0.99	0.99	0.93	0.95
7	F1sp	Mean	0.95	0.82	1.00	0.97	0.98	0.96
8	F2sp	Max	1.00	0.87	1.00	0.99	1.00	0.96
9	P2s	Max	0.96	0.92	1.00	1.00	0.95	0.92
10	P2i	Max	0.99	0.96	0.95	0.98	0.99	0.97
11	P1i	Max	0.99	0.96	0.98	0.99	0.99	0.98
12	P1s	Max	0.97	0.98	0.99	0.97	0.99	0.99
13	T2sp	Max	0.97	0.83	0.99	0.94	0.98	0.81
14	T3sp	Max	1.00	0.88	1.00	0.99	1.00	0.97
15	T1sp	Max	1.00	0.85	0.99	0.96	0.99	0.96
16	T2sa	Max	0.95	0.81	0.91	0.82	0.93	0.77
17	T3sa	Max	0.99	0.91	0.99	0.98	1.00	0.98
18	T1sa	Max	0.99	0.85	0.99	0.97	1.00	0.97
19	T2ia	Max	0.96	0.95	1.00	1.00	0.99	0.99
20	T3ia	Max	0.96	0.89	0.98	0.98	0.99	0.98
21	T1ia	Max	0.93	0.75	0.97	0.95	0.98	0.97
22	T2ip	Max	0.96	0.91	1.00	1.00	0.98	0.99
23	T3ip	Max	0.97	0.81	1.00	0.99	0.99	0.99
24	T1ip	Max	0.99	0.82	0.99	0.99	0.99	0.97

All SPECT/CT images were of sufficient quality to identify the necessary anatomical landmarks such as medial and lateral epicondyles, proximal tibia as well as femoral and tibial components. The measurements of femoral and tibial UKA component alignment were possible in all cases with sufficient definition of all axes. Although metal artifacts were present in nearly all cases, it did not cause a problem for UKA component or bone tracer uptake analysis.

For measurement of UKA component position there was strong agreement between the readings of the two observers; the ICC for the orientation of the femoral and tibial UKA component is presented in Table 2. The ICC for measurement of tibiofemoral angle was 0.96 (intra-observer reliability) and 0.99 (inter-observer reliability).

**Table 2:** **Inter- and intra-observer reliability (intra class correlation- ICC) for femoral, tibial and combined femoral-tibial component position after UKA**

			**Intraobserver-reliability**		**Interobserver-reliability**
**Measurement**	**Mean**	**SD**	**U1**	**U2**	**U1-U2**
**Femoral component**					
Varus-valgus	2.3	3.8	0.80	0,92	0.92
Flexion-extension	15.3	6.5	0.92	0.91	0.91
Internal rotation- external rotation	−4.3	5.0	0.74	0.92	0.92
**Tibial component**					
Varus-valgus	7.0	3.6	0.94	0.95	0.95
Flexion-extension	−6.3	5.0	0.98	0.99	1.00
Internal rotation- external rotation	−4.7	3.5	0.87	0.91	0.91
**Combined femoral and tibial component**					
Internal rotation- external rotation	10.2	7.1	0.93	0.95	0.95
**Tibiofemoral angle**					
Tibiofemoral angle	−1.7	2.8	0.98	0.99	0.98

## Discussion

The standard diagnostic work-up of patients with pain after UKA includes a detailed history and clinical examination [38]. Radiographs represent the first line imaging for routine assessment of UKA component position, loosening, fractures or infection [39]. However, the sensitivity of radiographs is limited as these are prone to projection errors [39]. Only clearly loose as well as strongly malpositioned UKA components can be identified [40,41].

For more subtle structural changes CT is the second line imaging. Here, the problem of projection error is overcome. Due to the metal components of knee arthroplasty CT is affected by metal artifact splatter. A problem, which has only partially been solved by metal artifact reduction software yet [42].

MRI is an adjunct imaging in patients with UKA, as these contain less metal parts than TKA [22,43]. It is helpful to identify OA disease progression, ligamentous lesions or soft tissue impingement. Currently its clinical value is limited, although first studies have shown promising results mainly in patients with prosthesis made of oxinium and not cobalt chrom, which is the most often used prosthesis material [22,43].

In contrast to solely morphological imaging such as CT and MRI nuclear bone imaging is more sensitive, as change of bone turnover occurs early in the disease process. Although SPECT/CT potentially can be very helpful in patients with pain after arthroplasty, it is to date not considered an essential part of the diagnostic work-up [44]. This is mainly due to the poor clinical evidence to support its common use.

However, recently the diagnostic value of SPECT/CT in patients with problems after knee surgery have been highlighted [23,25,27-35]. In particular the combination of information on mechanical alignment, component position and bone tracer uptake distribution leads to improved sensitivity and specificity in patients after knee arthroplasty [23,25,27,28,30-32,34,35]. The most important findings of our study were a nearly perfect inter- and intra-observer reliability for the position of UKA components in 3D-CT and determination of SPECT/CT tracer uptake (intensity and anatomical distribution pattern). The most striking advantage of SPECT/CT and our proposed method for the assessment of patients after UKA is, that if offers analysis of in vivo biomechanics in one imaging modality. Based on our localization scheme and analysis method we conceptionally strive to identify typical patterns of BTU distribution and intensity, which relate to typical pathologies such as femoral or tibial loosening, type and design of the UKA and/or mechanical alignment. Clearly, a cemented UKA will show different bone tracer uptake patterns than an uncemented one. A perfectly positioned UKA will show different BTUs than a varus tibial UKA component. In addition, the whole leg axis before and after surgery should to be taken into consideration. An increased tibial periprosthetic BTU in a medial UKA of a patient with a varus leg axis is more likely a normal bone remodeling than UKA loosening. The final diagnosis has to be guided by the CT findings. An overdiagnosis of loosening in patients after UKA can be the consequence, when only BTU patterns are considered and information on UKA position and alignment is neglected.

Using the approach of specific pathology related bone tracer uptake patterns a current dogma of nuclear bone imaging, which is that within the first 12 months physiological periprosthetic bone turnover leads to increased tracer uptake, might be overcome in future.

However, in patients after UKA assessment of BTU alone without evaluation of UKA position and alignment is nothing. The accurate determination of UKA component position in 3D reconstructed CT is a conditio sine qua non when aiming for identification of malposition of the UKA components [26,45]. Measurements of UKA component alignment (varus-valgus, internal rotation-external rotation, flexion-extension and tibial slope) in 3D reconstructed CT images, which are inherent part of the SPECT/CT data, offer an additional important benefit to the surgeon. These measurements of UKA component position can also be performed from 3D reconstructed CT alone [26]. However, then no correlation with bone tracer uptake patterns can be assessed.

Currently, analysis of SPECT/CT in patients after knee arthroplasty is still difficult. A close collaboration among orthopaedic surgeons and nuclear medicine physicians is the key factor to consider the necessary clinical information and SPECT/CT findings and guide optimal treatment. However, this interdisciplinary team approach is time consuming and highly dependent on available knowledge and resources. Many institutions lack this interdisciplinary setting. There is a high probability that quality of reporting and establishment of diagnosis in these institutions is disappointing for patients and their orthopaedic surgeons. Also a steep learning curve need to be considered here. Therefore, the orthopaedic and nuclear medicine fraternities should work together on improvement and standardization of their protocols and imaging analysis tools. Using specific analysis software and protocols as in this study could at least partly help to overcome the aforementioned limitations.

Based upon our findings, we propose the use of SPECT/CT including the presented algorithm and analysis methods in studies dealing with persistent or recurrent pain after UKA. Further clinical studies will reveal the clinical value of SPECT/CT for patients with problems after UKA.

## Conclusions

The SPECT/CT algorithm presented combining the mechanical information on UKA component position, alignment and metabolic data is highly reliable and proved to be a valuable, consistent and useful tool for analysing postoperative knees after UKA. Using this standardized approach in clinical studies might be helpful in establishing the diagnosis in patients with pain after UKA.

## Abbreviations

SPECT: Single photon emission computerized tomography
UKA: Unicondylar knee arthroplasty
CT: Computerized tomography
ICC: Intraclass correlation coefficients

## References

[1] Svard UC, Price AJ (2001). Oxford medial unicompartmental knee arthroplasty. A survival analysis of an independent series. J Bone Joint Surg Br.

[2] Saccomanni B (2010). Unicompartmental knee arthroplasty: a review of literature. Clin Rheumatol.

[3] Price AJ, Waite JC, Svard U (2005). Long-term clinical results of the medial Oxford unicompartmental knee arthroplasty. Clin Orthop Relat Res.

[4] Price AJ, Svard U (2011). A second decade lifetable survival analysis of the Oxford unicompartmental knee arthroplasty. Clin Orthop Relat Res.

[5] Biswal S, Brighton RW (2010). Results of unicompartmental knee arthroplasty with cemented, fixed-bearing prosthesis using minimally invasive surgery. J Arthroplasty.

[6] Song MH, Kim BH, Ahn SJ, Yoo SH, Shin SH (2010). Preventing lateral skin numbness after medial unicompartmental knee arthroplasty. Clin Orthop Surg.

[7] Song MH, Kim BH, Ahn SJ, Yoo SH, Lee MS (2009). Early complications after minimally invasive mobile-bearing medial unicompartmental knee arthroplasty. J Arthroplasty.

[8] Scott RD (2006). Three decades of experience with unicompartmental knee arthroplasty: mistakes made and lessons learned. Orthopedics.

[9] Saenz CL, McGrath MS, Marker DR, Seyler TM, Mont MA, Bonutti PM (2010). Early failure of a unicompartmental knee arthroplasty design with an all-polyethylene tibial component. Knee.

[10] Mariani EM, Bourne MH, Jackson RT, Jackson ST, Jones P (2007). Early failure of unicompartmental knee arthroplasty. J Arthroplasty.

[11] Bhutta MA, Doorgakant A, Marynissen H (2010). Tibial nerve impingement secondary to posterior cement extrusion after unicompartmental knee arthroplasty. J Arthroplasty.

[12] Springer BD, Scott RD, Thornhill TS (2006). Conversion of failed unicompartmental knee arthroplasty to TKA. Clin Orthop Relat Res.

[13] Saragaglia D, Estour G, Nemer C, Colle PE (2009). Revision of 33 unicompartmental knee prostheses using total knee arthroplasty: strategy and results. Int Orthop.

[14] McAuley JP, Engh GA, Ammeen DJ (2001). Revision of failed unicompartmental knee arthroplasty. Clin Orthop Relat Res.

[15] Jarvenpaa J, Kettunen J, Miettinen H, Kroger H (2010). The clinical outcome of revision knee replacement after unicompartmental knee arthroplasty versus primary total knee arthroplasty: 8–17 years follow-up study of 49 patients. Int Orthop.

[16] Jeer PJ, Mahr CC, Keene GC, Oakeshott RD (2006). Single photon emission computed tomography in planning unicompartmental knee arthroplasty. A prospective study examining the association between scan findings and intraoperative assessment of osteoarthritis. Knee.

[17] Rea P, Short A, Pandit H, Price AJ, Kyberd P, Beard DJ (2007). Radiolucency and migration after Oxford unicompartmental knee arthroplasty. Orthopedics.

[18] Fisher DA, Watts M, Davis KE (2003). Implant position in knee surgery: a comparison of minimally invasive, open unicompartmental, and total knee arthroplasty. J Arthroplasty.

[19] Soavi R, Loreti I, Bragonzoni L, La Palombara PF, Visani A, Marcacci M (2002). A roentgen stereophotogrammetric analysis of unicompartmental knee arthroplasty. J Arthroplasty.

[20] Aliprandi A, Sconfienza LM, Randelli P, Bandirali M, Tritella S, Di Leo G (2011). Magnetic resonance imaging of the knee after medial unicompartmental arthroplasty. Eur J Radiol.

[21] Aliprandi A, Perona F, Bandirali M, Randelli P, Cabitza P, Sardanelli F (2009). MR imaging of the knee in patients with medial unicompartmental arthroplasty: comparison among sequences at 1.5 T. Radiol Med.

[22] Heyse TJ, Figiel J, Hahnlein U, Timmesfeld N, Schmitt J, Schofer MD (2012). MRI after unicondylar knee arthroplasty: the preserved compartments. Knee.

[23] Hirschmann MT, Schon S, Afifi FK, Amsler F, Rasch H, Friederich NF (2013). Assessment of loading history of compartments in the knee using bone SPECT/CT: a study combining alignment and 99mTc-HDP tracer uptake/distribution patterns. J Orthop Res.

[24] Hirschmann MT, Wagner CR, Rasch H, Henckel J (2012). Standardized volumetric 3D-analysis of SPECT/CT imaging in orthopaedics: overcoming the limitations of qualitative 2D analysis. BMC Med Imaging.

[25] Hirschmann MT, Konala P, Iranpour F, Kerner A, Rasch H, Friederich NF (2011). Clinical value of SPECT/CT for evaluation of patients with painful knees after total knee arthroplasty–a new dimension of diagnostics?. BMC Musculoskelet Disord.

[26] Hirschmann MT, Konala P, Amsler F, Iranpour F, Friederich NF, Cobb JP (2011). The position and orientation of total knee replacement components: a comparison of conventional radiographs, transverse 2D-CT slices and 3D-CT reconstruction. J Bone Joint Surg Br.

[27] Hirschmann MT, Iranpour F, Konala P, Kerner A, Rasch H, Cobb JP (2010). A novel standardized algorithm for evaluating patients with painful total knee arthroplasty using combined single photon emission tomography and conventional computerized tomography. Knee Surg Sports Traumatol Arthrosc.

[28] Hirschmann MT, Mathis D, Rasch H, Amsler F, Friederich NF, Arnold MP (2013). SPECT/CT tracer uptake is influenced by tunnel orientation and position of the femoral and tibial ACL graft insertion site. Int Orthop.

[29] Mucha A, Dordevic M, Testa EA, Rasch H, Hirschmann MT (2013). Assessment of the loading history of patients after high tibial osteotomy using SPECT/CT--a new diagnostic tool and algorithm. J Orthop Surg Res.

[30] Hirschmann MT, Mathis D, Afifi FK, Rasch H, Henckel J, Amsler F (2013). Single photon emission computerized tomography and conventional computerized tomography (SPECT/CT) for evaluation of patients after anterior cruciate ligament reconstruction: a novel standardized algorithm combining mechanical and metabolic information. Knee Surg Sports Traumatol Arthrosc.

[31] Hirschmann MT, Davda K, Rasch H, Arnold MP, Friederich NF (2011). Clinical value of combined single photon emission computerized tomography and conventional computer tomography (SPECT/CT) in sports medicine. Sports Med Arthrosc.

[32] Hirschmann MT, Davda K, Iranpour F, Rasch H, Friederich NF (2011). Combined single photon emission computerised tomography and conventional computerised tomography (SPECT/CT) in patellofemoral disorders: a clinical review. Int Orthop.

[33] Konala P, Iranpour F, Kerner A, Rasch H, Friederich NF, Hirschmann MT (2010). Clinical benefit of SPECT/CT for follow-up of surgical treatment of osteochondritis dissecans. Ann Nucl Med.

[34] Hirschmann MT, Iranpour F, Davda K, Rasch H, Hugli R, Friederich NF (2010). Combined single-photon emission computerized tomography and conventional computerized tomography (SPECT/CT): clinical value for the knee surgeons?. Knee Surg Sports Traumatol Arthrosc.

[35] Hirschmann MT, Adler T, Rasch H, Hugli RW, Friederich NF, Arnold MP (2010). Painful knee joint after ACL reconstruction using biodegradable interference screws- SPECT/CT a valuable diagnostic tool? A case report. Sports Med Arthrosc Rehabil Ther Technol.

[36] Henckel J, Richards R, Lozhkin K, Harris S, Baena FM, Barrett AR (2006). Very low-dose computed tomography for planning and outcome measurement in knee replacement. The imperial knee protocol. J Bone Joint Surg Br.

[37] Walter SD, Eliasziw M, Donner A (1998). Sample size and optimal designs for reliability studies. Stat Med.

[38] Hofmann S, Seitlinger G, Djahani O, Pietsch M (2011). The painful knee after TKA: a diagnostic algorithm for failure analysis. Knee Surg Sports Traumatol Arthrosc.

[39] Sarmah SS, Patel S, Hossain FS, Haddad FS (2012). The radiological assessment of total and unicompartmental knee replacements. J Bone Joint Surg Br.

[40] Rasch H, Falkowski AL, Forrer F, Henckel J, Hirschmann MT (2013). 4D-SPECT/CT in orthopaedics: a new method of combined quantitative volumetric 3D analysis of SPECT/CT tracer uptake and component position measurements in patients after total knee arthroplasty. Skeletal Radiol.

[41] Hirschmann MT, Henckel J, Rasch H (2013). SPECT/CT in patients with painful knee arthroplasty-what is the evidence?. Skeletal Radiol.

[42] Huang JY, Kerns JR, Nute JL, Liu X, Balter PA, Stingo FC (2015). An evaluation of three commercially available metal artifact reduction methods for CT imaging. Phys Med Biol.

[43] Heyse TJ, le Chong R, Davis J, Boettner F, Haas SB, Potter HG (2012). MRI analysis for rotation of total knee components. Knee.

[44] Goergen TG, Dalinka MK, Alazraki N, Berquist TH, Daffner RH, DeSmet AA (2000). Evaluation of the patient with painful hip or knee arthroplasty. American College of Radiology. ACR Appropriateness Criteria. Radiology.

[45] Hirschmann MT, Mathis D, Afifi FK, Rasch H, Henckel J, Amsler F (2013). 4D-SPECT/CT in orthopaedics- combined quantitative volumetric 3D analysis of SPECT/CT tracer uptake and component position measurements in patients after total knee arthroplasty. Skeletal Radiol.

